# Hyperactivity in ADHD: Friend or Foe?

**DOI:** 10.3390/brainsci14070719

**Published:** 2024-07-17

**Authors:** Beverly-Ann Hoy, Michelle Bi, Matthew Lam, Gayuni Krishnasamy, Androu Abdalmalak, Barbara Fenesi

**Affiliations:** 1 Faculty of Education, Western University, London, ON N6G 1G7, Canada; bhoy6@uwo.ca (B.-A.H.); mbi5@uwo.ca (M.B.); mlam324@uwo.ca (M.L.); gkrish3@uwo.ca (G.K.); 2 Department of Physiology and Pharmacology, Western University, London, ON N6A 5C1, Canada; aabdalma@uwo.ca

**Keywords:** ADHD hyperactivity, ADHD hypofrontality, hypoarousal, ADHD fNIRS, physical activity

## Abstract

Background: Hyperactivity may play a functional role in upregulating prefrontal cortical hypoarousal and executive functioning in ADHD. This study investigated the neurocognitive impact of movement during executive functioning on children with ADHD. Methods: Twenty-four children with and without ADHD completed a Stroop task and self-efficacy ratings while remaining stationary (Stationary condition) and while desk cycling (Movement condition). Simultaneous functional near-infrared spectroscopy (fNIRS) recorded oxygenated and deoxygenated changes in hemoglobin within the left dorsolateral prefrontal cortex (DLPFC). Results: Among children with ADHD, the Movement condition produced superior Stroop reaction time compared to the Stationary condition (*p* = 0.046, *d* = 1.00). Self-efficacy improved in the Movement condition (*p* = 0.033, *d* = 0.41), whereas it did not in the Stationary condition (*p* = 0.323). Seventy-eight percent of participants showed greater oxygenation in the left DLPFC during the Movement condition vs. the Stationary condition. Among children without ADHD, there were no differences in Stroop or self-efficacy outcomes between Stationary and Movement conditions (*p*s > 0.085, *t*s < 1.45); 60% of participants showed greater oxygenation in the left DLPFC during the Movement vs. the Stationary condition. Conclusions: This work provides supportive evidence that hyperactivity in ADHD may be a compensatory mechanism to upregulate PFC hypoarousal to support executive functioning and self-efficacy.

## 1. Introduction

Attention-deficit hyperactivity disorder (ADHD) is the most common neurodevelopmental disorder, impacting 6% of children globally [1,2,3]. ADHD manifests through three primary symptoms: inattention, impulsivity, and hyperactivity [2,3,4]. These symptoms often emerge in preschool years and frequently persist into adulthood [5]. Children with ADHD often encounter difficulties in executive functioning (EF), which are higher-level cognitive functions essential for goal-directed, adaptive, and flexible behaviour [2]. EF involves three central processes: (1) inhibition—the control of attention and suppression of predominant responses; (2) switching—shifting attention from one task to another; and (3) working memory—retaining and processing information [3]. Children with ADHD tend to perform worse on EF tasks compared to their typically developing peers, and this often translates to greater academic challenges [4].

The EF deficits in ADHD, have, in part, been attributed to hypofrontality, which is a state of decreased cerebral blood flow in the prefrontal cortex (PFC) and correlated with executive dysfunction [6]. Children with ADHD exhibit distinct hypoactivation during EF tasks; electroencephalogram (EEG) studies demonstrate that PFC hypoactivation is consistently linked to increased slow wave (theta) activity and decreased fast wave (beta) activity during EF tasks [7]. This activity is involved in the frontoparietal and ventral attentional network, an area critical for EF [2]. Functional magnetic resonance imaging (fMRI) studies suggest that underlying hypofrontality is a diminished hemodynamic response, resulting in slower and less efficient delivery of blood, oxygen, and nutrients to active neuronal tissues [8]. These studies show ADHD-related hypoactivation localized to regions within the frontostriatal, frontoparietal, and ventral attention networks in comparison to controls [3,9]. Functional-near infrared spectroscopy (fNIRS) studies have similarly shown a diminished hemodynamic response especially within the left dorsolateral PFC (DLPFC) in those with ADHD in response to completing inhibitory control tasks (i.e., Stroop, n-back) and coincided with poorer task performance compared to those without ADHD [8,10,11,12].

Interestingly, increased gross motor activity (i.e., whole body movement requiring large muscles) during attention-demanding tasks may help improve hypofrontality associated with ADHD and promote EF [2,7,13]. Recent work found that higher rates of gross motor activity positively correlated with working memory performance for children with ADHD, but not for typically developing children [13]. Similarly, Ruiter et al. [2] found that adolescents with ADHD performed better on difficult trials of a working memory task if they were allowed to complete the task while using a desk cycle compared to their peers without ADHD. In addition, Hartanto et al. [14] showed that correct answers on an inhibitory control task were associated with more gross motor activity (i.e., more fidgeting) than incorrect answers. These findings are countercultural and counterintuitive as the predominant Western belief is that excessive gross motor activity, also known as “hyperactivity”, interferes with a child’s ability to learn; thus, educational settings mandate long periods of sedentarism during instruction. However, for children with ADHD, hyperactivity during tasks that require focused attention may be facilitative rather than harmful for their EF and therefore ability to learn [13].

This assertion is supported by extensive research showing that ADHD hypofrontality can be improved through intentional engagement in gross motor activity, otherwise known as physical activity. Both chronic and acute bouts of physical activity are effective for improving EF in younger and older adults [10,12,15,16,17,18], as well as children with and without ADHD [3,19,20,21,22,23], particularly in the domains of inhibitory control, working memory, decision making, and cognitive flexibility. Improvement in EF following repeated short bouts of physical activity may be connected to brain structural and functional changes, through increased cortical thickness and gray matter volume, as well as improved connectivity and neural activity [24,25]. Acute physical activity has also been shown to enhance the catecholamine system (e.g., dopamine and norepinephrine), leading to better attentional focus [21,26], as well as upregulating the release of brain-derived neurotrophic factor (BDNF), which aids synaptic formation and maturation, neurogenesis, stem cell activity, and neuronal survival [27,28]. Thus, physical movement, whether acute or chronic, has profound effects on the structure and function of the brain, and has been repeatedly shown to aid in enhancing EF in diverse populations.

Furthermore, the EF impairments evident in ADHD can offset mental processes that are important for learning, such as self-efficacy [1,23]. Self-efficacy refers to the belief an individual holds about their ability to successfully perform a specific task or behaviour [29]. Students who score higher on self-efficacy measures have more confidence to set higher goals and can self-regulate and monitor their impulses while facing difficult tasks [30]. This often leads to better performance in academic settings. Individuals with ADHD often exhibit lower self-efficacy ratings, which may play a role in their poor academic performance [31,32]. Physical activity and self-efficacy are closely connected [22,33] such that habitual physical activity is linked to greater adaptable cognitive reappraisal strategies when dealing with stressful events [34]. This suggests that physical activity has the potential to improve one’s ability to manage difficult tasks, which is a skill in self-efficacy that individuals with ADHD often find challenging.

Taken all together, there is an extensive body of literature that supports the link between acute physical activity, EF, and self-efficacy, as well as an emerging field of research linking hyperactivity to improved hypofrontality and EF in children with ADHD. The current study used behavioural and neuroimaging techniques to further elucidate the impact of movement during EF on PFC activation, inhibitory control, and self-efficacy in children with and without ADHD. We aimed to answer the following research questions:

1. How does movement during an EF task impact performance in children with and without ADHD?
2. How does movement during an EF task impact left DLPFC activity in children with and without ADHD?
3. How does movement during an EF task impact self-efficacy in children with and without ADHD?

We hypothesized that (1) movement would improve inhibitory control in children with and without ADHD, with a greater magnitude of benefit among those with ADHD [14,20,35]; (2) movement would increase left DLPFC activation more than a stationary condition, especially for those with ADHD [12]; and (3) movement would increase self-efficacy in children with and without ADHD, with a greater magnitude of benefit for those with ADHD [22,33,34].

## 2. Materials and Methods

### 2.1. Participants

A total of 30 children aged 8–12 were recruited for the study based on a sample size calculation using G*Power (version 3.1.9.6) with a medium-to-large effect size Cohen’s d = 0.90 [13], power of 0.95, alpha of 0.05, with the primary outcome variable being executive functioning in a repeated measures within–between interaction. Four participants provided incomplete data, and two were extreme outliers during the Stroop task (SPSS step of 1.5 × interquartile range). To avoid potential bias in missing data, these participants were removed from the final data set. Thus, 24 children aged 8–12 comprised the final sample size, with 15 in the control group (mean age = 10.3; females = 5) and 9 in the ADHD group (mean age = 9.8; females = 2). Participants were recruited through flyers posted in London’s Children’s Museum, the Institution’s Mary J. Wright Child and Youth Development Clinic, as well as through community centers throughout the city. Flyers were also emailed out to a list of current clients (guardians and children) at the Mary J. Wright Child and Youth Development Clinic who previously indicated that they would be interested in participating in research studies. A telephone/email recruitment script was used, depending on the contact method they provided. Inclusion criteria for the ADHD group were children between the ages of 8 and 12 with a suspected or formal diagnosis of ADHD by a physician or psychologist, who were English literate (speaking and reading). Inclusion criteria for the non-ADHD group (controls) were the same as the ADHD group but without the ADHD diagnosis. Participants were excluded if they were not fully literate or did not speak English, if they had comorbid disorders such as epilepsy, autism, or intellectual disabilities, if they were unable to participate in moderate-intensity physical activity, and if they were colour-blind.

### 2.2. Measures

#### 2.2.1. Demographic Questionnaire

Table 1 provides demographic information derived from a questionnaire that was completed by the participants’ guardians. Questions included the guardians’ age, sex, level of education, current employment status, and income. It also included questions relating to the participants’ age, sex, ADHD diagnosis status, other medical diagnoses, prescribed medication, and history of medication.

#### 2.2.2. Vanderbilt ADHD Diagnostic Parent Rating Scale

Table 2 provides data from the Vanderbilt scale. Guardians completed the Vanderbilt Parent Rating Scale (VADPRS) to verify their child’s ADHD status [36]. The VADPRS includes all 18 of the DSM-5 criteria for ADHD. Additionally, it screens for comorbidities such as oppositional-defiant disorder, conduct disorder, and anxiety and depression. The tool asks guardians to rate the severity of behaviours on a 4-point scale, ranging from 0 (never) to 3 (very often). In addition to the behavioural questions asked, the performance section of the VADPRS is an 8-item scale with four items related to academic performance: (a) overall academic performance, (b) reading, (c) mathematics, and (d) written expression; and another four items included to evaluate relationships with (e) peers, (f) siblings, (g) parents, and (h) participation in organized activities. Guardians’ rate each of these on a 5-point scale from “problematic” to “above average”. To meet the DSM-5 criteria for an ADHD diagnosis, the child must score at least a 2 (often) or 3 (very often) on 6 out of 9 inattentive or hyperactive core symptoms, or both, and score a 4 on at least 2, or 5 on at least 1 of the impairment questions. The VADPRS is a validated measure found to have both high reliability and clinical utility [37].

#### 2.2.3. Executive Functioning: Stroop Task

The Stroop task is commonly used to measure inhibitory control in children with ADHD [38,39]. A keyboard version of the Stroop task was employed, whereby specific keyboard keys were assigned to represent certain colours, presented on a 24-inch computer monitor. The assigned keys included “D” (representing “red”), “F” (representing “green”), “J” (representing “blue”), and “K” (representing “black”). Coloured stickers were placed on the assigned keys to enable participants to visually see the appropriate key. Participants were presented with words spelling out various colours; the words were either printed in the same colour that the word implies (congruent) or a different colour that the word implies (incongruent). For example, during congruent tasks, the word “red” would be printed in the colour red; the correct answer would be red. In incongruent trials, the word “red” would be printed in the colour “blue”, for example; in this case, the correct answer would be blue. Participants were required to press on the corresponding key to make a response. Participants were encouraged to go as fast as possible and to avoid making errors, as the response times and accuracy (correct vs. incorrect) answers were recorded. The task was designed in Inquisit (version 6.6.0) and was programmed to have 4 blocks of the Stroop task (50 s each) with 20 s rest periods in between each block.

#### 2.2.4. Self-Efficacy

Self-efficacy was measured using an adapted version of the “Self-Efficacy” scale developed by Bandura and Wessels [40]. The assessment is used to measure how confident one is in their ability to do well on a task. The 11-point Likert scale ranges from 0 (“not at all”) to 10 (“completely”). Participants completed the scale at the beginning and end of each experimental block for a total of 4 times throughout the study (i.e., pre-movement Stroop, post-movement Stroop; pre-stationary Stroop, post-stationary Stroop).

#### 2.2.5. Heart Rate

To track exercise intensity, heart rate was continuously measured with an Apple Watch and the corresponding VeryFit 2.0 app, that was connected to an iPhone. The Apple Watch was placed on the right wrist of all participants and tested to ensure consistent and accurate readings before the experimental protocol began. The “Workout” feature was chosen as the physical activity category on the VeryFit app. Heart rate values during the Movement and Stationary conditions were dynamically sent from the watch to the app, and average heart rate was recorded using the app data and recorded after each condition. The Apple Watch uses an accelerometer and photoplethysmography (PPG) sensor, which continuously measures heart rate during physical activity by using green LED lights paired with light-sensitive photodiodes to calculate beats per minute [41,42]. If the signal is weak, the heart rate sensor increases the LED brightness and sampling rate to obtain accurate readings [41,42]. The Apple Watch has been shown to provide the most accurate measure of heart rate compared to other physical activity wearable devices [42,43]. The VeryFit app is compatible with both iOS devices and syncs seamlessly with the Apple Watch, allowing for real-time heart rate monitoring.

#### 2.2.6. Left DLPFC Activity Using Functional Near-Infrared Spectroscopy (fNIRS)

The left DLPFC was selected as the region of interest (ROI) based on past literature investigating the effects of exercise on Stroop Task performance [10,12]. Activity within this ROI was recorded using fNIRS—a non-invasive, safe, and portable functional neuroimaging tool, often recommended to utilize in young participants [44]. The NIRSsport version of the fNIRS system used in the current study is suitable for experiments involving movement or physical activity [45]. fNIRS measures changes in oxygenated (HbO) and deoxygenated (HbR) hemoglobin concentrations in the blood, which indirectly relate to brain activity [43]. fNIRS measures neurovascular coupling that takes place in the brain; when the brain is active, it consumes oxygen and, as a result, blood flow is increased towards the activated tissue [44,46].

The fNIRS cap consisted of 8 sources (dual wavelength of 760 and 830 nm) and 7 detectors forming an 18-channel montage spanning the prefrontal cortex. The source-detector distance across all channels were at least 3 cm, and the sampling rate was 10.2 Hz. Montage creation and fNIRS data collection were conducted using NIRx’s software NIRSite (version 2021.4) and Aurora (version 2021.4), respectively. Signal quality of the channels was assessed using the coefficient of variation metric (CV) and signal levels. Abiding by NIRx recommendations, the coefficient of variation metric (CV) levels were maintained below 3% and signal levels were at least 3 mV (excellent reading). During experimentation, a cap (EASYCAP, GmbH) was used to secure the infrared-emitting optodes to the scalp. Infrared light emitted through the skin and skull from the “source” optodes and were absorbed by brain tissue. The change in HbO and HbR was then processed by a nearby “detector” optode based on the degree of light absorption. The change in hemoglobin concentration was subsequently calculated via the Beer–Lambert law, which assesses the shift in intensity of the emitted light in conjunction with the received light [44].

#### 2.2.7. Design

This study utilized a within-subjects counterbalanced design wherein each participant participated in both the Movement and Stationary condition in a random order.

##### Movement Condition

**Figure 1:**
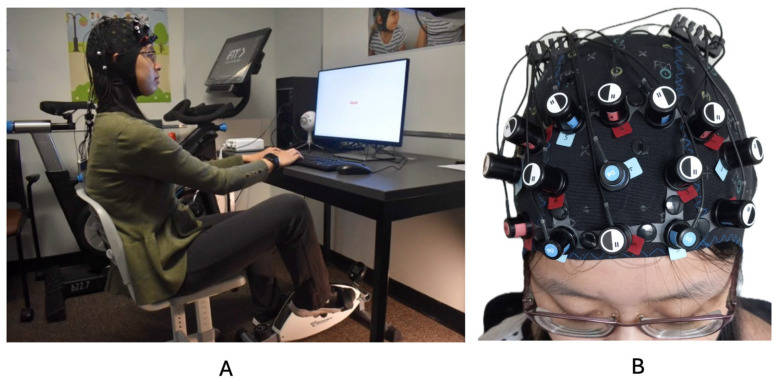
(**A**) depicts a representative participant and the experimental setup; (**B**) depicts fNIRS cap setup.

A desk cycle (3D Innovations Desk Cycle) was used to enable movement during completion of the desk-based Stroop task [2,24]. Several participant-specific adjustments were made for each experimental session to ensure participants could easily pedal while also sitting comfortably at the desk station: the height of the chair, the distance away from the desk and keyboard, and the bike pedals. Participants’ feet were strapped into the pedals of the desk cycle and the cycling resistance was set to the lowest setting. Participants were encouraged to pedal at a consistent non-exertive pace throughout the experimental protocol. If they pedaled too fast or too slow, they were verbally encouraged to increase or decrease their pedaling speed. The fNIRS cap remained on for the duration of the experiment. The average heart rate during the Movement condition for those with ADHD was 93 bpm (SD = 7), and for Controls, it was 95 bpm (SD = 8). Figure 1 depicts the experimental setup.

##### Stationary Condition

Participants remained stationary with their feet on the floor during the completion of the Stroop task, self-efficacy ratings, and fNIRS recording. The average heart rate during the Stationary condition for those with ADHD was 85 bpm (SD = 12), and for Controls, it was 89 bpm (SD = 8).

##### Procedure

This study was conducted at the Faculty of Education building at Western University. All participants were asked to abstain from ADHD medication use for 24 h prior to commencing the study. Participants had the option to decline the requirement, with their decision being recorded. Guardians and participants were greeted by a researcher and were guided through consent and assent forms. If participants agreed to participate, then the study proceeded. While the guardian completed the VADPRS and demographic questionnaire, the child participant was brought into an adjacent room and familiarized with the study protocol. Participants were introduced to the fNIRS equipment and had the opportunity to look at the fNIRS cap and touch the infrared optodes to get comfortable. Any questions or concerns were addressed. The circumference of each participant’s head was measured using fabric measuring tape, and based on the measurement, an appropriately sized fNIRS cap was selected. The cap was positioned according to the 10–20 electrode system to ensure consistent placement between participants [47]. Participants were seated while researchers placed the cap on their head. Participants were asked to sit still for approximately 30 s while optimization of the fNIRS channels was completed by the researchers, ensuring that the CV levels were below 3% and signal levels were at least 3 mV. After the optimization process, researchers showed participants what their brain activity looked like on the display screen.

**Figure 2:**
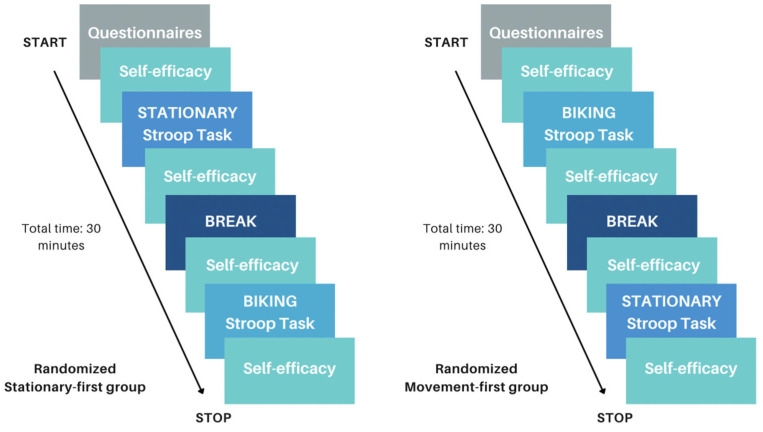
This figure shows the flow of the experimental procedure. The left depicts the experimental procedure if a participant was randomized to perform the Stationary condition first; the right depicts the experimental procedure if a participant was randomized to perform the biking Movement condition first.

Figure 2 provides a flow diagram of the study procedure. Participants were provided instructions about the Stroop task and self-efficacy ratings and had the opportunity to complete several practice Stroop trials before beginning the real task. All participants completed both Stationary and Movement conditions. Conditions were counterbalanced such that participants were randomly assigned to perform the Stationary or Movement condition first. In the Movement condition, participants were asked to cycle on the desk cycle for 1 min to familiarize themselves with the apparatus and to find a stable, manageable pace. In the Stationary condition, participants began the experiment immediately. Participants completed four blocks of the Stroop task (50 s each) with 20 s rest periods between each task-block. At the beginning and the end of each condition, participants were asked to rate their self-efficacy for the Stroop task. At the end of the study, participants and their guardians were debriefed, and researchers answered any remaining questions.

### 2.3. Statistical Analysis

#### 2.3.1. Executive Functioning and Self-Efficacy

All statistical analyses for EF and self-efficacy outcomes were conducted using SPSS 29. For EF outcomes, two 2 × 2 mixed factorial analyses of variance (ANOVA) were conducted for Stroop RT and Stroop proportion correct outcomes with a within-subject factor of condition (Movement vs. Stationary) and a between-subject factor of group (ADHD vs. Control). Exploratory paired samples *t*-tests were performed to compare within-participant differences in outcome measures between movement and stationary conditions separately for ADHD and Control groups. For self-efficacy, two 2 × 2 mixed factorial ANOVA were conducted for pre–post changes in self-efficacy ratings in the Stationary and Movement conditions separately. Exploratory paired-samples *t*-tests were conducted to compare within-participant changes in self-efficacy pre and post each condition separately for ADHD and Control groups.

#### 2.3.2. fNIRS and Left DLPFC Activity

fNIRS data were preprocessed and analyzed using Satori (v 2.06). First, the raw data were trimmed by 10 s before the first and after the last trigger. The raw data were then converted into optical density and corrected for motion artifacts using spike removal and TDDR [48]. Next, the optical density data were converted into changes in concentration using the modified Beer–Lambert law [49] and band-pass filtered ([0.01–0.5 Hz]) to remove high-frequency noise. Lastly, the signals were normalized, and general linear modeling (GLM) analysis was conducted at the individual level. For each participant, the contrast of movement > stationary was investigated. The left DLPFC locations on the fNIRS montage are the regions F1, F3, F5 (channels S4-D2, S1-D2, and S1-D1, respectively). Paired samples *t*-tests were conducted to compare HbO and HbR differences in ROI channels between Movement and Stationary conditions. Significant activation of at least one channel within the ROI was considered a marker of ROI activation.

## 3. Results

### 3.1. Preliminary Analyses

To check for potential confounding effects of counterbalancing the order of the Movement and Stationary conditions, two 2 × 2 mixed factorial ANOVA were conducted on Stroop RT and proportion correct scores where condition order (Stationary-first, Movement first) was included in addition to the factors of the main analysis. Results showed no significant effects of counterbalancing on RT or proportion correct (*F*s < 2.03, *p*s > 0.17), indicating that counterbalancing order did not impact the effect of our experimental manipulation.

### 3.2. Executive Functioning: Stroop

Table 3 provides EF descriptives for both ADHD and Control groups. For Stroop RT, the 2 × 2 mixed factorial ANOVA yielded a main effect of condition F(1,22) = 5.87, *p* = 0.024, ηp^2^ = 0.21, no main effect of group F(1,22) = 0.20, *p* = 0.659, ηp^2^ = 0.01, and no interaction F(2,22) = 0.47, *p* = 0.503, ηp^2^ = 0.02. Exploratory paired *t*-test analyses within the ADHD group yielded superior Stroop RT in the Movement condition compared to the Stationary condition t(8) = 1.91, *p* = 0.046, d = 1.00. Within the Control group, there was no difference between the Movement and Stationary condition on Stroop RT t(14) = 1.45, *p* = 0.085. For Stroop proportion correct, the 2 × 2 mixed factorial ANOVA yielded no main effect of condition F(1,22) = 0.001, *p* = 0.994, ηp^2^ = 0.00, no main effect of group F(1,22) = 0.01, *p* = 0.931, ηp^2^ = 0.00, and no interaction F(2,22) = 0.01, *p* = 0.929, ηp^2^ = 0.00. Exploratory paired *t*-test analyses within the ADHD and Control groups yielded similar Stroop proportion correct in Movement and Stationary conditions (all *t*s < 0.074, all *p*s > 0.471).

### 3.3. fNIRS: Left DLPFC Oxygenation

**Figure 3:**
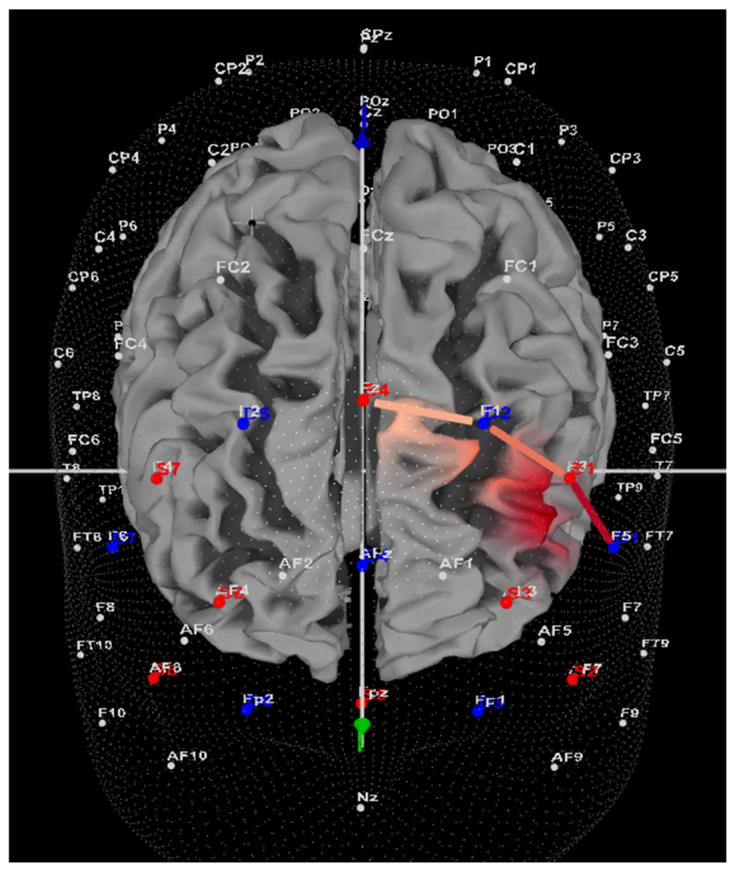
This is a representative image of a participant with ADHD when comparing concentrations of oxygenated hemoglobin in the left DLPFC between the Movement and Stationary conditions. The red shade on the right (left hemisphere) indicates greater oxygenated hemoglobin in the regions of interest, i.e., F1, F3 and F5, corresponding to the left DLPFC. This image is masked to only show channels over the ROI.

Within the ADHD group, seven of nine participants (78%) showed greater HbO during the Movement condition compared to the Stationary condition in the left DLPFC (all *t*s > 5.4, all *p*s < 0.001). Please refer to Supplementary Tables S1 and S2 for a full breakdown of fNIRS *t*-test results. Only two of nine participants (22%) showed greater HbO during the Stationary condition in the left DLPFC (all *t*s < −3.25, all *p*s < 0.001). Figure 3 provides a representative image of a participant with ADHD and their HbO concentration differences during Movement vs. Stationary conditions while performing the Stroop task. Within the Control group, 9 of 15 participants (60%) showed greater HbO during the Movement condition compared to the Stationary condition in the left DLPFC (all *t*s > 2.16, all *p*s < 0.03). In comparison, 6 of 15 participants (40%) showed greater HbO during the Stationary condition in the left DLPFC (all *t*s < −2.5, all *p*s < 0.01).

### 3.4. Self-Efficacy

Table 4 provides self-efficacy descriptives for both ADHD and Control groups. Within the Movement condition, the 2 × 2 factorial ANOVA yielded no main effect of time F(1,22) = 1.92, *p* = 0.181, ηp^2^ = 0.084, no main effect of group F(2,22) = 0.083, *p* = 0.776, ηp^2^ = 0.004., and no interaction F(1,22) = 1.92, *p* = 0.181, ηp^2^ = 0.084. Within the Stationary condition, the 2 × 2 factorial ANOVA yielded no main effect of time F(1,22) = 1.14, *p* = 0.30, ηp^2^ = 0.049, no main effect of group F(1,22) = 0.004, *p* = 0.95, ηp^2^=, and no interaction F(2,22) = 0.36, *p* = 0.553, ηp^2^ = 0.016. Exploratory paired *t*-test analyses within the ADHD group yielded a significant increase in self-efficacy for the Stroop task during the Movement condition t(8) = 2.14, *p* = 0.033, d = 0.41, but not in the Stationary condition t(8) = 0.48, *p* = 0.323. Exploratory paired *t*-test analyses within the Control group yielded no change in self-efficacy from pre-post Stroop task during the Movement or Stationary conditions (all ts < 0.48, *p*s > 0.323).

### 3.5. Missing Cases

Four participants provided incomplete data. Differences in these cases compared to those that did not have missing data were assessed along the variables of age, sex, age diagnosed with ADHD, ADHD subtype, and self-efficacy scores in the Movement and Stationary conditions. There was a technical issue with the computer software delivering the Stroop task, so all executive functioning data were missing. Importantly, these missing data were not due to participants not being willing to provide responses but due to technical issues, thus limiting systematic bias. Among the four excluded participants (compared to the complete data set), there were no differences (*p* > 0.05) in average age (incomplete data: M = 9.2 yrs, SD = 1.8 vs. complete data: M = 9.8 yrs, SD = 1.7), with differences (*p* < 0.05) among the variables of child sex (incomplete data: 67% male vs. Complete data: 78%), age diagnosed (incomplete data: 100% at 7 yrs old vs. complete data: 22% at 7 yrs old), and ADHD subtype (incomplete data: 100% combined subtype vs. complete data: 44% combined subtype). For self-efficacy ratings, there were no differences in the Stationary condition pre-Stroop or post-Stroop ratings (pre-incomplete data: M = 8, SD = 1.7 vs. pre-complete data: M = 7.22, SD = 2.5; post-incomplete data: M = 6.5 (1.5) vs. post-incomplete data: M = 7.44, SD = 3.3), or in the Movement condition post-Stroop ratings (incomplete data: M = 8.5 (SD = 1) vs. complete data: M = 8.3, SD = 2.1). There was a significant difference among the self-efficacy variables of the Movement pre-Stroop self-efficacy ratings (incomplete data: M = 2.6 SD = 0.5 vs. complete data: M = 7.4, SD = 2.3). Given the pattern of the complete data, even if the Movement pre-Stroop self-efficacy ratings were included, they would follow the same pattern as the complete data, and even potentially inflate the pairwise differences. Although some missing cases were different from complete cases, a complete case analysis with removal of incomplete cases was the selected approach given the missing executive functioning values, which was a primary outcome variable. We recognize that this approach reduced our sample size, and the limitations of this has been included below.

## 4. Discussion

The current study found that movement during a cognitively demanding task, compared to remaining stationary, may support inhibitory control and self-efficacy for children with ADHD. Not only did children with ADHD show improved RT on a Stroop task if able to desk cycle; they also showed increased neural activation within the left DLPFC, a region often showing reduced activity in those with ADHD. In comparison, children without ADHD performed similarly well whether engaged in movement or remaining stationary during the Stroop task. However, children without ADHD also showed increased activation in the left DLPFC during the Movement condition compared to the Stationary condition, although this did not translate to significantly greater inhibitory control outcomes. Implications for how we view hyperactivity in ADHD, as well as study limitations, are discussed below.

The most salient finding from the current work is that movement during the completion of an inhibitory control task had evident neurocognitive benefits for children with ADHD. This finding is in line with our hypotheses and previous research with children and adolescents who show improved working memory and inhibitory control when using a desk cycle [2], when engaging in more gross motor activity [13], or when engaging in spontaneous motor activity [14]. For adolescents with ADHD, movement was only beneficial for working memory during high-demand (i.e., difficult) trials compared to remaining stationary [2]. However, in the current study, children with ADHD benefitted from movement during an inhibitory control task regardless of task difficulty, suggesting that movement during EF tasks may have a greater magnitude of impact for younger individuals. Children with ADHD have higher rates of activity and fidgeting compared to those without ADHD [50], and this has traditionally been viewed as an impairment. However, an emerging theory is that such increased movement may serve as a compensatory mechanism to augment chronic hypofrontality associated with ADHD [51]. Indeed, the fNIRS findings from the current study lend support to the benefit of movement for PFC functioning and corresponding improvements in inhibitory control. Specifically, children with ADHD had greater left DLPFC oxygenation (i.e., more efficient influx of nutrient and resource-rich blood into neural tissue) when engaging in movement during the Stroop task compared to when remaining stationary. These results lend both behavioural and neuroscientific support to the emerging model that predicts a positive relation between movement/gross motor activity and EF for individuals with ADHD. Thus, hyperactivity may be helpful in compensating for hypoarousal among children with ADHD to improve cognitive functioning.

Movement during EF also improved self-efficacy for children with ADHD. Self-efficacy is tightly linked to academic achievement. Children with ADHD not only report lower self-efficacy but also often struggle academically compared to their typically developing peers [52]. In line with our predictions, the current study showed that self-efficacy in performing the Stroop task significantly improved during the Movement condition for children with ADHD, while the same increase in self-efficacy was not evident during stationary Stroop performance. Our previous work similarly showed that physical activity improves both self-efficacy and inhibitory control using a flanker task among typically developing children and youth [53]. With a larger sample size, mediation analyses could be conducted to provide a more descriptive relationship between movement, self-efficacy, and inhibitory control; in other words, does movement improve self-efficacy for cognitively challenging tasks, and does this, in turn, improve performance? Importantly, higher scores on self-efficacy scales have been linked to the ability to self-regulate and monitor impulses in the face of challenging tasks, thus directly impacting EF and ultimately learning.

Movement during EF did not improve inhibitory control or self-efficacy for children without ADHD. This result is in line with several previous studies failing to find a beneficial effect of active workstations on EF among typically developing individuals [54,55]. This prior research suggests that undertaking physical activity such as walking on a treadmill while performing a simple finger-tapping task may lead to a decrement in performance due to attentional interference [56]. Two additional explanations offer diametrically opposed views, with both employing the optimal stimulation model consisting of an inverted-U shape function between arousal and performance. On the one hand, children without ADHD may require minimal to no physical exertion during EF to reach optimal performance as they do not necessarily experience cerebral hypoactivation. On the other hand, the desk cycling movement in the current study may not have reached sufficient intensity to support improved inhibitory control. Prior research has shown that individuals without ADHD who participated in acute high-intensity physical activity showed higher left DLPFC activation alongside improved Stroop performance [57]. Interestingly, in the current study, children without ADHD did show increased left DLPFC activation during the Movement condition compared to the Stationary condition (60% of controls). However, this increase in neural activity did not translate to improved inhibitory control; this more likely points to insufficient physical activity intensity rather than attentional interference; otherwise, the Stationary condition would likely have yielded superior Stroop performance.

Findings from this work highlight the importance of revisiting how we view and often condemn “hyperactivity” in children with ADHD. Engaging in movement during attention demanding tasks may be functional for some children and may support their ability to learn. Long periods of sedentary behaviour are detrimental for EF across diverse populations [58] but especially for those who may require more movement to augment aspects of their neurodiversity, such as reduced PFC activity. This has particular implications for classroom environments where seated learning is an expectation; this may contribute to diminished learning opportunities for children with ADHD as they may need more stimulation to perform at a level similar to their neurotypical peers. Classroom management is always challenging, and it is undeniable that encouraging children to remain attentive and on-task is no easy feat for teachers; however, overregulating gross motor movement (i.e., hyperactivity) may be detrimental to some children. Furthermore, it may be beneficial to provide devices such as desk cycles or activity balls or techniques such as active learning lessons [59,60] that can be incorporated into the classroom with minimal disruption. Indeed, future work should evaluate what other stimuli beyond desk cycles may support PFC activation and executive functioning in both children with and without ADHD and work well with classroom management strategies. Additional work in this field should consider examining how ADHD subtype (inattention, hyperactivity, combined), as well as ADHD severity, impacts the results, as these individual difference factors may play a role in the type of support that children require. Most importantly, the narrative around hyperactivity as a negative and disruptive component of ADHD may be more accurately explained as the individuals’ attempt to upregulate their central nervous system and reap effective EF outcomes.

### Limitations

The sample size in the ADHD group was smaller than originally anticipated due to incomplete data provided by some participants. However, an examination of previous studies using fNIRS on participants with ADHD shows that sample sizes of as small as seven can yield reliable effects [61]. In addition, desk cycling was self-paced, and cycling intensity varied slightly across participants. We purposefully did not control for cycling intensity to allow each participant to titrate the intensity according to their own intrinsic sense of stimulation need. It is important to note that most participants were on ADHD medication. Although most participants refrained from using medication 24 h prior to the study, it is possible that those on medication differed in their performance compared to those not on medication. However, prior research involving physical activity paradigms has shown that medication use does not impact the responsiveness of children with ADHD to physical activity interventions. Participants were not screened for psychiatric disorders beyond the exclusion criteria, nor were common comorbidities such as oppositional defiant disorder (ODD) controlled for; thus, comorbidities may have had unknown influences on cognitive functioning and potentially impacted findings. Unfortunately, these disorders are commonly comorbid with ADHD; thus, it would be exceptionally challenging to recruit ADHD-exclusive diagnoses. Additionally, four out of the nine ADHD participants’ VADPRS results did not match their formal diagnosis. However, we relied on the parent-reported diagnosis as it considers more holistic information and thus still categorized these children as having ADHD; DSM-compliant diagnosis requires reports from two settings (teacher/school and parent/guardian), whereas the VADPRS only considers parent/guardian reports. Although the VADPRS is considered a psychometrically sound assessment tool, some research suggests the VADPRS alone only predicts ADHD diagnosis at approximately 56% [62]. Lastly, it would have been beneficial to include more than one measure of inhibitory control in addition to the Stroop task. Multiple tasks tapping the same construct but differing in other aspects (i.e., design, modality) would provide a more robust outcome measure compared to using a single task alone [2].

## 5. Conclusions

This work provides supportive evidence for the emerging theory that hyperactivity in ADHD may serve as an unconscious compensatory mechanism by which to upregulate chronic hypoarousal, especially within the PFC, to support EF. This study uniquely combined behavioural evidence in the form of inhibitory control and self-efficacy measures with neuroscientific evidence in the form of neural measures within the left DLPFC to demonstrate the benefit of movement during EF among children with ADHD. Taken all together, this research provides further evidence that strongly encourages the reframing of hyperactivity as a maladaptive and harmful behaviour to an adaptative and supportive behaviour for neurodiverse children who may require different levels of stimulation to succeed.

## Figures and Tables

**Table 1:** Demographic information for participants and their guardians.

Characteristic	ADHD Value (%)	Control Value (%)
Age of Child (years), mean (SD)	9.8 (1.7)	10.3 (1.2)
Age of Guardian (years), mean (SD)	38.8 (3.8)	43.4 (4.9)
Sex of Participant		
Male	7 (78)	10 (67)
Female	2 (22)	5 (33)
Sex of Guardian		
Male	1 (11)	2 (13)
Female	8 (89)	13 (87)
Guardian’s Education Level (*n*)		
Some high school, no diploma	0 (0)	0 (0)
High school graduate, diploma or the equivalent	0 (0)	0 (0)
Some college credit, no degree	0 (0)	2 (13)
Trade/technical/vocational training	0 (0)	1 (7)
Associate degree	1 (11)	1 (7)
Bachelor’s degree	4 (44)	5 (33)
Master’s degree	2 (22)	4 (27)
Professional degree	1 (11)	2 (13)
Guardian’s Employment (*n*)		
Employed for wages	3 (33)	6 (40)
Self-employed	3 (33)	6 (40)
Out of work	1 (11)	1 (7)
Homemaker	1 (11)	2 (13)
No response	1 (11)	-
Household Income		
Prefer not to say	2 (22)	3 (20)
<CAD 30,000	0 (0)	1 (7)
CAD 30,000–CAD 40,000	0 (0)	1 (7)
CAD 40,000–CAD 50,000	0 (0)	0 (0)
CAD 50,000–CAD 60,000	1 (11)	4 (27)
CAD 60,000–CAD 70,000	1 (11)	2 (13)
CAD 70,000–CAD 80,000	0 (0)	1 (7)
CAD 80,000–CAD 90,000	2 (22)	1 (7)
CAD 90,000–CAD 100,000	1 (11)	2 (13)
>CAD 100,000	2 (22)	0 (0)
Age of ADHD diagnosis (*n*)		-
Unsure	1 (11)	-
3	3 (33)	-
7	2 (22)	-
8	1 (11)	-
9	1 (11)	-
10	1 (11)	-
ADHD Subtype (*n*)		-
Predominantly inattentive	2 (22)	-
Predominantly hyperactive	0 (0)	-
Combined subtype	4 (44)	-
Unsure/no diagnosis given	3 (33)	-
Currently Taking ADHD Medication		-
No response	1 (11)	-
Yes	6 (67)	-
No	2 (22)	-
Other Diagnosis Present		-
Yes	3 (33)	-
No	6 (67)	-
Medicated for Another Diagnosis		-
Yes	3 (33)	-
No	5 (56)	-
No Response	1 (11)	-

**Table 2:** Vanderbilt Parent Rating Scale.

	ADHD *N* (%)	Control *N* (%)
Predominantly Inattentive subtype		
Clinically significant	2 (22)	0 (0)
Not clinically significant	7 (78)	15 (100)
Predominantly Hyperactive/Impulsive subtype		
Clinically significant	1 (11)	0 (0)
Not clinically significant	8 (89)	15 (100)
ADHD Combined Inattention/Hyperactivity		
Clinically significant	2 (22)	0 (0)
Not clinically significant	7 (78)	15 (100)
Oppositional-defiant disorder		
Clinically significant	3 (33)	0 (0)
Not clinically significant	6 (67)	15 (100)
Conduct disorder		
Clinically significant	1 (11)	0 (0)
Not clinically significant	8 (89)	15 (100)
Anxiety		
Clinically significant	2 (22)	0 (0)
Not clinically significant	7 (78)	15 (100)
Impairment		
Clinically significant	6 (67)	1 (7)
Not clinically significant	3 (33)	14 (93)

**Table 3:** Descriptive statistics for Stroop task across conditions and groups.

Outcome Variables	ADHD (*N* = 9)		Control (*N* = 15)	
	Stationary Mean (SD)	Movement Mean (SD)	Stationary Mean (SD)	Movement Mean (SD)
Stroop RT (ms)	1268.40 (439.70)	935.11 (170.07)	1141.62 (459.97)	964.75 (181.22)
Stroop Proportion Correct	0.89 (0.14)	0.89 (0.16)	0.90 (0.12)	0.90 (0.14)

**Table 4:** Descriptive statistics for self-efficacy outcomes across conditions and groups.

Condition	ADHD (*N* = 9)		Control (*N* = 15)	
	Self-Efficacy Pre-Stroop Mean (SD)	Self-Efficacy Post-Stroop Mean (SD)	Self-Efficacy Pre-Stroop Mean (SD)	Self-Efficacy Post-Stroop Mean (SD)
Stationary	7.22 (2.54)	7.44 (3.25)	6.87 (2.9)	7.67 (2.02)
Movement	7.11 (3.62)	8.33 (2.06)	7.43 (2.31)	7.43 (2.41)

## Data Availability

The data presented in this study are available upon request from the corresponding author. The data are not publicly available due to ethical restrictions.

## Supplementary Materials

The following supporting information can be downloaded at https://www.mdpi.com/article/10.3390/brainsci14070719/s1: Table S1: fNIRS GLM contrast results for the ADHD group; Table S2: fNIRS GLM contrast results for the Control group.
