# Supplementary material for: Hyperactivity in ADHD: Friend or Foe?
Source: Brain Sci. 2024 Jul 17;14(7):719. doi: 10.3390/brainsci14070719 (PMC11274564; doi:10.3390/brainsci14070719)
Supplement: Supplementary file 1 [file brainsci-14-00719-s001.zip › Table S1.pdf]

Table S1. fNIRS GLM contrast results for ADHD group

Participant ID	DLPFC ROI	HbO		HbR	
		T-value	P-value	T-value	P-value
	F1	-4.93	<.001	11.32	<.001
2	F3	5.45	<.001	-15.14	<.001
	F5	10.36	<.001	-9.06	<.001
	F1	12.71	<.001	-5.75	<.001
13	F3	18.36	<.001	3.16	.002
	F5	25.12	<.001	-7.6	<.001
	F1	-21.78	<.001	-1.86	.06
3	F3	5.4	<.001	-.25	.80
	F5	-13.09	<.001	-11.33	<.001
	F1	9.31	<.001	13.93	<.001
5	F3	9.84	<.001	-.12	.90
	F5	6.01	<.001	1.61	.11
	F1	-4.24	<.001	-11.52	<.001
17	F3	-6.65	<.001	-8.12	<.001
	F5	7.68	<.001	-12.82	<.001
	F1	1.19	.23	-1.09	.27
24	F3	6.61	<.001	4.96	<.001
	F5	5.71	<.001	16	<.001
	F1	17.01	<.001	11.12	<.001
14	F3	12.76	<.001	14.41	<.001
	F5	-7.14	<.001	9.21	<.001
	F1	-11.84	<.001	11.66	<.001
9	F3	-13.27	<.001	12.31	<.001
	F5	-7.3	<.001	-.15	.88
	F1	-1.5	.12	32	<.001
27	F3	.86	.38	16	<.001
	F5	-3.25	.001	-3.8	<.001

Note. Grey shading denotes when the Movement condition produced significantly greater HbO than the Stationary condition.
