# Supplementary material for: Hyperactivity in ADHD: Friend or Foe?
Source: Brain Sci. 2024 Jul 17;14(7):719. doi: 10.3390/brainsci14070719 (PMC11274564; doi:10.3390/brainsci14070719)
Supplement: Supplementary file 1 [file brainsci-14-00719-s001.zip › Table S2.pdf]

Table S2. fNIRS GLM contrast results for Control group

Participant ID	DLPFC ROI	HbO		HbR	
		T-value	P-value	T-value	P-value
	F1	4	<.001	-7	<.001
4	F3	14	<.001	-8	<.001
	F5	4	<.001	9	<.001
	F1	-3	.004	2.4	.01
11	F3	-13	<.001	1.5	.11
	F5	7	<.001	12	<.001
	F1	14	<.001	-9	.06
15	F3	-12	<.001	17	.80
	F5	9	<.001	-15	<.001
	F1	5.4	<.001	-.3	.77
18	F3	-1.6	.11	12	.90
	F5	15	<.001	9	<.001
	F1	-10	<.001	17	<.001
20	F3	-19	<.001	35	<.001
	F5	-1.6	.1	7.1	<.001
	F1	-2.5	.01	16	<.001
7	F3	-4	<.001	-2.5	.01
	F5	-16	<.001	11	<.001
	F1	2.16	.03	5.7	<.001
10	F3	-17	<.001	2.2	.02
	F5	5.3	<.001	.5	.59
	F1	-25	<.001	14	<.001
16	F3	-12	<.001	-8	<.001
	F5	-24	<.001	-13	<.001
	F1	-27	<.001	19	<.001
23	F3	-2.7	.01	12	<.001
	F5	-15	<.001	10	<.001
	F1	4.5	<.001	2.9	.004
22	F3	9.1	<.001	-12.7	<.001
	F5	4.2	<.001	3.7	<.001
	F1	-4.9	<.001	-11.3	<.001
6	F3	-1.7	.09	4.3	<.001
	F5	2.4	.02	-5.7	<.001
	F1	9.1	<.001	3.7	<.001
8	F3	6.4	<.001	-18.2	<.001
	F5	20.1	<.001	-5.8	<.001
	F1	3.5	<.001	.8	.4
12	F3	-2.1	.03	-.7	.5

	F5	3.9	<.001	-26.9	<.001
	F1	16.9	<.001	12.09	<.001
25	F3	5.4	<.001	-9.2	<.001
	F5	-4.7	<.001	12.2	<.001
	F1	-7	<.001	-12	<.001
26	F3	-14.7	<.001	-6.9	<.001
	F5	-13.1	<.001	-3	<.001

Note. Grey shading denotes when the Movement condition produced significantly greater HbO than the Stationary condition.
